# Clinical features of COVID-19-related optic neuritis: a retrospective study

**DOI:** 10.3389/fneur.2024.1365465

**Published:** 2024-04-12

**Authors:** Fang-Fang Zhao, Yun Wang, Tai-Ping Li, Shuan Hu, Xin-Sheng Yu, Xinxin Li, Jingyun Cen, Kefan Huang, Hongjie Lin, Jian-Feng Yang, Lan Chen, Ling-Ping Cen

**Affiliations:** ^1^Joint Shantou International Eye Center of Shantou University and The Chinese University of Hong Kong, Shantou, Guangdong, China; ^2^Shantou University Medical College, Shantou, Guangdong, China; ^3^Shaoguan University Medical College, Shaoguan, Guangdong, China

**Keywords:** optic neuritis associated with COVID-19 (COVID-19 ON), clinical features, best-corrected visual acuity (BCVA), orbital magnetic resonance imaging (MRI), glucocorticoid therapy

## Abstract

**Objective:**

This retrospective study aimed to investigate the clinical features of optic neuritis associated with COVID-19 (COVID-19 ON), comparing them with neuromyelitis optica-associated optic neuritis (NMO-ON), myelin oligodendrocyte glycoprotein-associated optic neuritis (MOG-ON), and antibody-negative optic neuritis (antibody-negative ON).

**Methods:**

Data from 117 patients (145 eyes) with optic neuritis at the Shantou International Eye Center (March 2020–June 2023) were categorized into four groups based on etiology: Group 1 (neuromyelitis optica-related optic neuritis, NMO-ON), Group 2 (myelin oligodendrocyte glycoprotein optic neuritis, MOG-ON), Group 3 (antibody-negative optic neuritis, antibody-negative ON), and Group 4 (optic neuritis associated with COVID-19, COVID-19 ON). Characteristics of T2 and enhancement in orbital magnetic resonance imaging (MRI) were assessed. Best-corrected visual acuity (BCVA) was compared before treatment, at a short-term follow-up (14 days), and at the last follow-up after treatment.

**Results:**

The COVID-19-associated optic neuritis (COVID-19 ON) group exhibited 100% bilateral involvement, significantly surpassing other groups (*P* < 0.001). Optic disk edema was observed in 100% of COVID-19 ON cases, markedly differing from neuromyelitis optica-related optic neuritis (NMO-ON) (*P* = 0.023). Orbital magnetic resonance imaging (MRI) revealed distinctive long-segment lesions without intracranial involvement in T1-enhanced sequences for the COVID-19 ON group compared to the other three groups (*P* < 0.001). Discrepancies in optic nerve sheath involvement were noted between the COVID-19 ON group and both NMO-ON and antibody-negative optic neuritis (antibody-negative ON) groups (*P* = 0.028). Before treatment, no significant difference in best-corrected visual acuity (BCVA) existed between the COVID-19 ON group and other groups. At the 14-day follow-up, BCVA in the COVID-19 ON group outperformed the NMO-ON (*P* < 0.001) and antibody-negative ON (*P* = 0.028) groups, with no significant difference observed compared to the myelin oligodendrocyte glycoprotein optic neuritis (MOG-ON) group. At the last follow-up after treatment, BCVA in the COVID-19 ON group significantly differed from the NMO-ON group (*P* < 0.001).

**Conclusion:**

Optic neuritis associated with COVID-19 (COVID-19 ON) predominantly presents with bilateral onset and optic disk edema. Orbital magnetic resonance imaging (MRI) demonstrates that COVID-19 ON presents as long-segment enhancement without the involvement of the intracranial segment of the optic nerve in T1-enhanced images. Glucocorticoid therapy showed positive outcomes.

## Introduction

Severe acute respiratory syndrome coronavirus 2 (SARS-CoV-2), the virus responsible for COVID-19, is well-documented for inducing significant pulmonary complications, including pneumonia and acute respiratory distress syndrome (ARDS). In addition to its pulmonary ramifications, clinicians have observed a spectrum of extrapulmonary manifestations associated with COVID-19. Notably, ocular manifestations, ranging from conjunctival congestion and conjunctivitis to more severe conditions such as keratoconjunctivitis, retinal changes, uveitis, and optic neuritis, have been reported in individuals with COVID-19 (1–11). However, the existing literature on optic neuritis associated with COVID-19 (COVID-19 ON) predominantly comprises case reports, lacking a comprehensive examination of its clinical characteristics.

Optic neuritis (ON), characterized by inflammation of the optic nerve, presents in various forms—autoimmune, infectious, or systemic. Autoimmune optic neuritis includes distinct subtypes such as neuromyelitis optica-related optic neuritis (NMO-ON), collapsin response mediator protein 5 optic neuritis (CRMP5-ON), myelin oligodendrocyte glycoprotein ON (MOG-ON), multiple sclerosis ON (MS-ON), single isolated optic neuritis (SION), relapsing isolated optic neuritis (RION), and chronic relapsing inflammatory optic neuropathy (CRION). Antibody-negative optic neuritis (antibody-negative ON) is classified into SION and RION based on the course of the disease. Systemic disorders causing optic neuritis include allergic granulomatous angiitis, anti-neutrophil cytoplasmic autoantibody (ANCA)-associated vasculitis, ankylosing spondylitis, Behçet's disease, Churg–Strauss disease, Cogan syndrome, giant cell arteritis, granulomatosis with polyangiitis, IgG 4 disease, Kawasaki disease, microscopic polyangiitis, polyarteritis nodosa, primary antiphospholipid syndrome, rheumatic disease, sarcoidosis, Sjögren syndrome, systemic lupus erythematosus, Susac's syndrome, systemic sclerosis, Takayasu arteritis, treatment side-effects, ulcerative colitis, and Wegener granulomatosis. COVID-19 infections can directly affect the optic nerve and may also trigger optic neuritis as a post-infectious or post-vaccination phenomenon. This trigger can result in infectious optic neuropathies, which is a less common but diverse group with varied and non-specific clinical presentations (12). Infectious optic neuropathies are less common and may present with varied, overlapping, and non-specific clinical appearances (13). COVID-19 ON falls within the category of infectious optic neuropathies. This retrospective study aims to comprehensively explore and compare the clinical features of COVID-19 ON with those of NMO-ON, MOG-ON, and antibody-negative ON.

## Materials and methods

### Study cohort

This retrospective study was conducted at the Joint Shantou International Eye Center of Shantou University and The Chinese University of Hong Kong between March 2020 and June 2023. All patients were treated with intravenous methylprednisolone (at a dose of 20 mg/kg/day for children and 1 g/day for adults) for 3–5 days, followed by a taper of oral prednisone (at a starting dose of 1 mg/kg/day) with variable durations, based on the subtype of ON and recovery from optic neuritis. Follow-up data were obtained during the return visit for clinical examinations. For patients whose follow-up compliance time is less than half a year, the recurrence status will be obtained by telephone follow-up with the patient or their guardian.

The diagnostic and inclusion criteria for patients with ON are as follows (12): (1) Monocular, subacute loss of vision associated with relative afferent pupillary defect (RAPD) and with or without orbital pain worsening on eye movements, (2) Binocular, subacute loss of vision associated with or without relative afferent pupillary defect (RAPD) or orbital pain worsening on eye movements, (3) Orbital magnetic resonance imaging (MRI): Contrast enhancement of the symptomatic optic nerve and sheaths acutely, with or without T2 hyperintensity within 3 months, (4) Biomarkers: Aquaporin 4 (AQP4) and MOG antibody seropositive, and (5) NMO-ON and MOG-ON conform to their respective diagnostic guidelines (14, 15). All patient sera were tested for autoantibodies, including ANCA, antinuclear antibody (ANA), human leukocyte antigen-B27 (HLA-B27), anti-Sjögren's syndrome-related antigen A (SSA), anti-Sjögren's syndrome-related antigen B (SSB), rheumatoid factor (RF), and antibodies related to tuberculosis, hepatitis B, syphilis, HIV, and COVID-19. The exclusion criteria for the study are as follows: (1) any other types of optic neuropathy, including compressive, vascular, toxic, metabolic, infiltrative, or hereditary optic neuropathy, (2) presence of craniocerebral lesions other than those from demyelinating diseases involving the optic chiasm or optic pathway downstream of the optic chiasm and the optic cortex, (3) glaucoma or any other ocular diseases that could influence visual acuity (VA), and (4) unknown serum MOG and AQP4 antibody status. The diagnostic and inclusion criteria for patients with COVID-19 ON are as follows (12): (1) diagnosis of optic neuritis, (2) exclusion of other types of infectious optic neuritis, (3) negative AQP4, and MOG antibodies, and (4) confirmed diagnosis of COVID-19 within the past 28 days, with current positivity for antibodies. The exclusion criterion for the study was the absence of acute clinical manifestations of new COVID-19.

A total of 117 patients (involving 145 eyes) were retrospectively categorized into four groups based on etiology: Group 1 comprised 45 patients (50 eyes) with neuromyelitis optica-related optic neuritis (NMO-ON); Group 2 included 14 patients (19 eyes) with myelin oligodendrocyte glycoprotein optic neuritis (MOG-ON); Group 3 consisted of 52 patients (64 eyes) with antibody-negative optic neuritis (antibody-negative ON); and Group 4 encompassed six patients (12 eyes) with COVID-19-associated optic neuritis (COVID-19 ON).

### Study protocol

All patients underwent a comprehensive ophthalmic evaluation, which included the assessment of Snellen best-corrected visual acuity (BCVA), slit-lamp examination, measurement of intraocular pressure (IOP), and Humphrey automated static perimetry for visual field assessment (VF). Dilated fundus photography and peripapillary retinal nerve fiber layers (pRNFLs) were assessed to evaluate optic disk swelling before treatment using high-definition spectral domain optical coherence tomography (SD-OCT: Carl Zeiss Meditec, Dublin, CA, USA). BCVA was examined by the standard table of vision logarithms at 5 m. Individuals who were unable to read any letter at a distance of 1 m were further examined by finger counts, hand movements, or perceiving light. Visual parameters, converted to logarithm of the minimum angle of resolution (logMAR) units for statistical analysis, were recorded at baseline, 14-day follow-up, and final follow-up visits. The onset of symptoms in both eyes within a span of 2 weeks is considered as simultaneous bilateral onset.

All patients underwent orbital magnetic resonance imaging (MRI) with fat-suppressed T2-weighted images and gadolinium-enhanced T1 sequences at a magnetic field strength of 1.5 T. The imaging encompassed the optic nerve and the optic chiasm, with axial scans precisely aligned to these structures. The slice thickness was 3 mm with a 0.5-mm interslice interval. In orbital MRI, the optic nerve was divided into three regions: orbital, canalicular, and intracranial. The lesion length or the optic nerve length was determined by multiplying the slice thickness (3 mm) and interslice interval (0.5 mm) by the number of coronal sections showing optic nerve changes on T1-enhanced images, with or without long T2 sequences. Long-segment lesions were those exceeding half the length of the optic nerve, as measured using MicroDicom Viewer software, version 3.4.7 (MicroDicom DICOM viewer Copyright). For the statistical analysis of orbital MRI, cases from external institutions with lesions that could not be measured for length were excluded. MRI of the head or spinal regions was performed on patients presenting with myelitis or systemic symptoms. The images were reviewed blindly by two independent raters (FFZ and LPC). When there was a mismatch between MRI findings, the images were reviewed by both readers and a consensus was reached.

### Statistical methods

Statistical analysis was carried out using SPSS software version 25.0 (IBM Co., Chicago IL). Quantitative data were expressed as mean ± standard deviation or median and range, as appropriate. Appropriate parametric and non-parametric tests, including the chi-squared test, analysis of variance (ANOVA), Fisher's exact test, the Mann–Whitney *U*-Test, and the Kruskal–Wallis test, were applied reasonably. Bonferroni-corrected pairwise comparisons were used to adjust for multiple comparisons. *P*-values of < 0.05 were considered statistically significant.

## Results

### Demographic and clinical features

**Figure 1:**
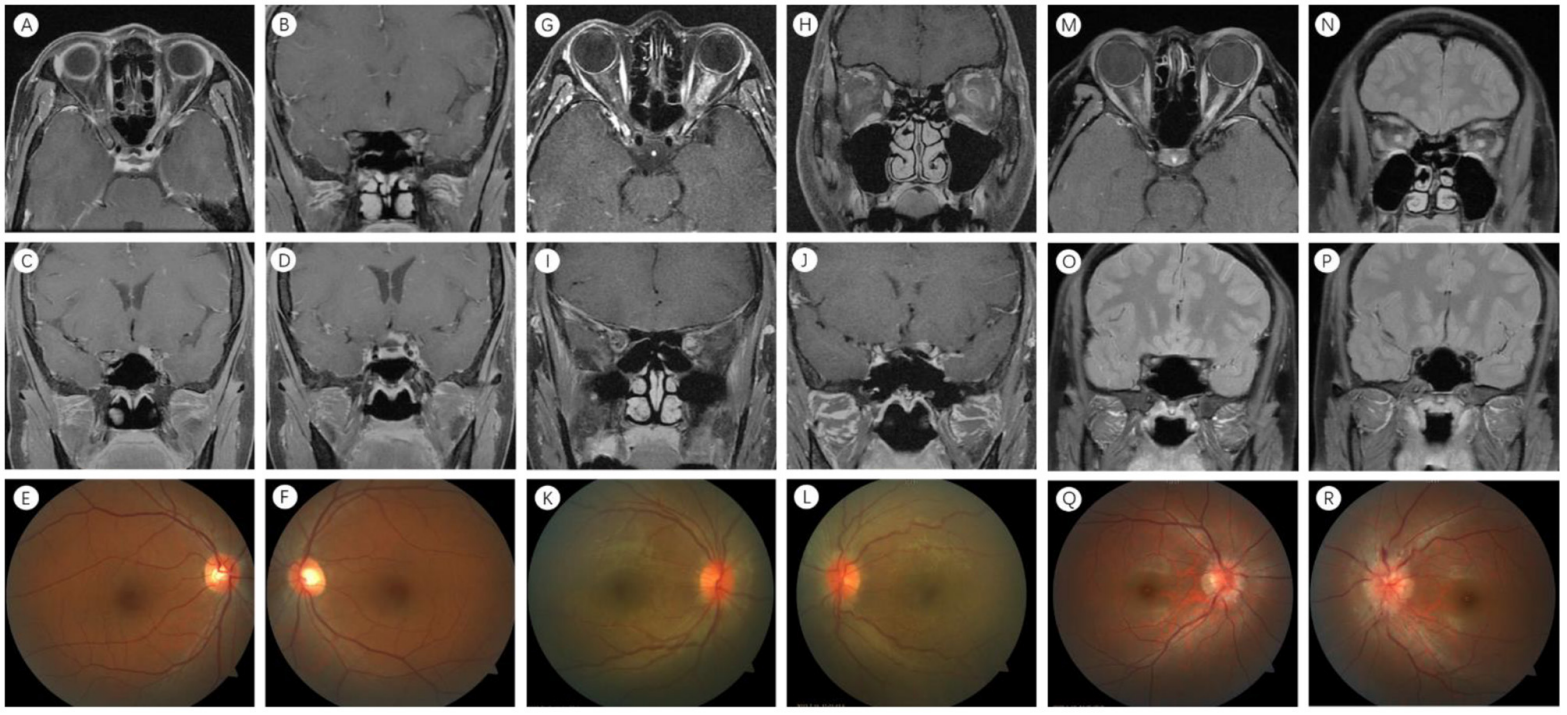
Neuroimaging features in NMO-ON, MOG-ON, and COVID-19 ON. **(A–F)** Left eye in NMO-ON. **(A)** Orbital MRI: orbital enhancement. **(B)** Orbital MRI: canalicular enhancement. **(C)** Orbital MRI: intracranial enhancement. **(D)** Orbital MRI: optic chiasm enhancement. **(E, F)** No abnormalities in bilateral optic nerve intraocular segments. **(G–L)** Left eye in MOG-ON. **(G)** Orbital MRI: orbital enhancement. **(H, I)** Orbital MRI: orbital enhancement with meningeal enhancement. **(J)** Orbital MRI: intracranial enhancement. **(K)** No abnormalities in the right optic nerve intraocular segment. **(L)** Left optic nerve intraocular segment swelling. **(M–R)** Both eyes in COVID-19-ON. **(M, N)** Orbital MRI: bilateral orbital enhancement. **(O)** Orbital MRI: bilateral canalicular enhancement. **(P)** Orbital MRI: no abnormal intracranial enhancement. **(Q, R)** Bilateral optic nerve intraocular segment swelling. NMO-ON, neuromyelitis optica-related optic neuritis; MOG-ON, myelin oligodendrocyte glycoprotein optic neuritis; COVID-19 ON, optic neuritis associated with COVID-19; MRI, magnetic resonance imaging.

The main demographic and clinical data of the study are presented in Table 1. Four groups were statistically similar regarding age, sex, time of onset, and the final follow-up time point. The mean ages of the patients were 40.2, 29.6, 34.4, and 30.5 years for the four groups (*p* = 0.115, Table 1), and female preponderance was observed in all four groups. The median time from onset was 10.0 days (range, 7.0–15.0 days) in Group 1, 10.0 days (range, 5.0–14.0 days) in Group 2, 10.0 days (range, 7.0–14.0 days) in Group 3, and 7.0 days (range, 7.0–9.0 days) in Group 4 (*p* = 0.358; Table 1). The corresponding values at the final follow-up point were 92.0 days (range, 18.5–258.5 days) in Group 1, 49.0 days (range, 18.5–187.5 days) in Group 2, 60.5 days (range, 17.8–199.3 days) in Group 3, and 42.0 days (range, 17.0–107.0 days) in Group 4 (*p* = 0.436; Table 1). Group 4 exhibited 100% bilateral involvement, which was significantly higher than the other groups (*P* < 0.001, Table 1). Optic disk edema was observed in 100% of Group 4, indicating a significant difference, compared to Group 1 (*P* = 0.023, Table 1; Figure 1). When compared to Group 3, Group 1 exhibited a predisposition to recurrence (*P* < 0.001, Table 1). In Group 3 (Antibody-negative ON group), two patients (3.8%) were classified as RION and one patient (1.9%) as CRION.

**Table 1:** Demographic and clinical details of patients in four groups of optic neuritis.

	**NMO-ON (*n* = 45, eye = 50)**	**MOG-ON (*n* = 14, eye = 19)**	**Antibody-negative ON (*n* = 52, eye = 64)**	**COVID-19 ON (*n* = 6, eye = 12)**	***P*-value**
Age (years)	40.2 ± 2.2	29.6 ± 4.3	34.4 ± 2.5	30.5 ± 7.5	0.115^$^
Sex (male/female)	8/37	4/10	19/33	1/5	0.2^*^
Bilateral, *n* (%)	5 (11.1)	5 (35.7)	12 (23.1)	6 (100)	< 0.001^*^
Optic disk edema, eyes (%)	28 (56)	12 (63.2)	46 (71.9)	12 (100)	0.023^‡^
Relapse, *n* (%)	18 (40)	1 (7.1)	3 (5.8)	0 (0)	0.029^*^
Time from onset, median (range day)	10 (7.0–15.0)	10 (5.0–14.0)	10 (7.0–14.0)	7 (7.0–9.0)	0.358^#^
Final follow-up (day)	92 (18.5–258.5)	49 (18.5–187.5)	60.5 (17.8–199.3)	42 (17.0–107.0)	0.436^#^

^*^Based on Fisher's exact test.

^$^Based on the chi-squared test.

^#^Based on the Kruskal–Wallis test.

^‡^Based on analysis of variance (ANOVA).

NMO-ON, neuromyelitis optica-related optic neuritis; MOG-ON, myelin oligodendrocyte glycoprotein optic neuritis; antibody-negative ON, antibody-negative optic neuritis; COVID-19 ON, optic neuritis associated with COVID-19.

### Visual acuity

The median baseline BCVA for Groups 1, 2, 3, and 4 was 1.85 logMAR (range, 1.62–2.30 logMAR), 1.85 logMAR (range, 0.81–2.45 logMAR), 1.52 logMAR (range, 0.67–1.85 logMAR), and 1.31 logMAR (range, 0.64–1.85 logMAR), respectively (*p* = 0.012; Table 2). After applying the Bonferroni correction for multiple comparisons, Group 1 showed worse vision compared to Group 3 (*p* = 0.026, Table 2). At the 14-day follow-up, the corresponding values for Groups 1, 2, 3, and 4 were 1.39 logMAR (range, 0.70–1.85 logMAR), 0.22 logMAR (range, 0.07–0.52 logMAR), 0.30 logMAR (range, 0.97–0.80 logMAR), and 0.00 logMAR (range, 0.00–0.19 logMAR), respectively (*p* < 0.001; Table 2). Furthermore, Group 1 exhibited the worst vision compared with other groups (*p* < 0.001), and Group 4 demonstrated better vision compared with Group 3 (*p* = 0.028). Four groups showed a statistical visual improvement from baseline to the 14-day follow-up (*p* < 0.001, *p* < 0.001, *p* < 0.001, and *p* = 0.005 for Group 1, Group 2, Group 3, and Group 4, respectively) (Table 2). At the final follow-up time, the corresponding values for Group 1, 2, 3, and 4 were 1.34 logMAR (range, 0.30–1.85 logMAR), 0.97 logMAR (range, 0.00–0.55 logMAR), 0.97 logMAR (range, 0.00–0.30 logMAR), and 0.00 logMAR (range, 0.00–0.97 logMAR), respectively (*p* < 0.001; Table 2). Finally, Group 1 exhibited the worst vision compared with other groups (*p* < 0.001). Four groups showed a statistical visual improvement from baseline to the final follow-up (*p* < 0.001, *p* < 0.001, *p* < 0.001, and *p* = 0.005 for Group 1, Group 2, Group 3, and Group 4, respectively) (Table 2). No serious adverse effects of steroid therapy were documented in this study.

**Table 2:** Best-corrected visual acuity (BCVA) during the course of the study for each group.

				**Groups**				***P*-value**
	**Time**		**Total**	**NMO-ON**	**MOG-ON**	**Antibody-negative ON**	**COVID-19 ON**	
BCVA	Baseline	Value	1.85 (0.82–2.30)	1.85 (1.62–2.30)	1.85 (0.81–2.45)	1.52 (0.67–1.85)	1.31 (0.64–1.85)	0.012^#^
	14-day follow-up (day)	Value	0.52 (0.97–1.52)	1.3 (0.70–1.85)	0.22 (0.071–0.52)	0.30 (0.97–0.80)	0.00 (0.00–0.19)	< 0.001^#^
		*P*-within		< 0.001^†^	< 0.001^†^	< 0.001^†^	0.005^†^	
Final follow-up (day)	Value	0.15 (0–1.40)	1.34 (0.30–1.85)	0.97 (0.00–0.55)	0.97 (0.00–0.30)	0.00 (0.00–0.97)	< 0.001^#^
	*P*-within		< 0.001^†^	< 0.001^†^	< 0.001^†^	0.005^†^	

^#^Based on the Kruskal–Wallis test.

^†^Based on the Mann–Whitney *U*-Test.

BCVA, best-corrected visual acuity; NMO-ON, neuromyelitis optica-related optic neuritis; MOG-ON, myelin oligodendrocyte glycoprotein optic neuritis; antibody-negative ON, antibody-negative optic neuritis; COVID-19 ON, optic neuritis associated with COVID-19.

### MRI manifestation

Cases from external institutions with lesions that could not be measured for length were excluded. A total of 99 eyes were retrospectively categorized into four groups: Group 1 comprised 35 eyes with NMO-ON; Group 2 included 10 eyes with MOG-ON; Group 3 consisted of 46 eyes with antibody-negative ON; and Group 4 encompassed 8 eyes with COVID-19 ON. Table 3 presents the results of optic nerve MRI, revealing T1-enhanced images with or without long T2 sequences. No significant differences in orbital, canalicular, intracranial, and long-segment lesions were observed among the four groups (*p* = 0.305, *p* = 0.712, *p* = 0.287, and *p* = 0.165) (Table 2). Regarding the involvement of the optic chiasm, Group 1 exhibited a significant difference compared to Group 3 (*p* = 0.032, Table 3). Concerning the involvement of the optic nerve sheath, differences were noted between Group 4 and both Group 1 and Group 3 (*p* = 0.028, Table 3). In terms of long-segment lesions not involving the intracranial segment, significant differences were observed between Group 4 and the other three groups (*p* < 0.001, Table 3; Figure 1).

**Table 3:** Comparison of the optic nerve MRIs in four groups.

	**NMO-ON**	**MOG-ON**	**Antibody-negative ON**	**COVID-19 ON**	***P*-value**
No. of eyes	35	10	46	8	
Orbital, eyes (%)	33 (92.3)	8 (80)	44 (95.7)	8 (100)	0.305^*^
Canalicular, eyes (%)	17 (48.6)	5 (50)	28 (60.9)	4 (50)	0.712^*^
Intracranial	14 (40)	5 (50)	22 (47.8)	1 (12.5)	0.287^*^
Chiasmal, eyes (%)	5 (14.3)	0 (0)	0 (0)	0 (0)	0.032^*^
The optic nerve sheath, eyes (%)	30 (8.6)	3 (30)	7 (15.2)	4 (50)	0.028^*^
Long-segment lesions, eyes (%)	17 (48.6)	4 (40)	27 (58.7)	7 (87.5)	0.165^*^
Long-segment not involving the intracranial segment, eyes (%)	5 (14.3)	1 (10)	6 (13)	7 (87.5)	< 0.001^*^

^*^Based on Fisher's exact test.

NMO-ON, neuromyelitis optica-related optic neuritis; MOG-ON, myelin oligodendrocyte glycoprotein optic neuritis; antibody-negative ON, antibody-negative optic neuritis; COVID-19 ON, optic neuritis associated with COVID-19; MRI, magnetic resonance imaging.

## Discussion

Our study investigated the clinical features of COVID-19 ON, categorizing it as an infectious optic neuropathy. The etiology of bacterial infectious optic neuropathies includes *Bartonella, Brucella, Coxiella burnetii*, leprosy, *Mycoplasma pneumoniae*, ocular cat-scratch disease, *Streptococcus*, syphilis, tuberculosis, Whipple disease, and typhus. Fungal infectious optic neuropathies are mainly associated with histoplasmosis, while parasitic infectious optic neuropathies are primarily linked to toxoplasmosis and neurotoxocarosis. The etiology of viral infectious optic neuropathies encompasses Chikungunya fever, cytomegalovirus, coronavirus, dengue, Epstein–Barr virus, echovirus, hepatitis B and C, herpes simplex, human immunodeficiency virus (HIV), human herpesvirus 6, Inoue-Melnick virus, measles, mumps, rubella, varicella-zoster virus (VZV), tick-borne encephalitis, West Nile virus, and Zika virus (12). COVID-19 belongs to the category of coronaviruses within the viral class.

Optic neuritis induced by cytomegalovirus, dengue, echovirus, hepatitis B, herpes simplex, human herpesvirus 6, measles, mumps, rubella, West Nile virus, and Zika virus is primarily reported in individual case studies, lacking a systematic summary of clinical presentations, MRI characteristics, and treatment efficacy. Limited reports on optic neuritis caused by HIV suggest that patients may experience unilateral or bilateral onset, often with the manifestation of optic disk swelling. The recommended treatment is directed toward addressing the underlying primary condition. Optic neuropathy related to HIV demonstrates axonal degeneration, possibly mediated by infected macrophages and increased expression of proinflammatory cytokines (16, 17). Another small number of reports on varicella-zoster virus-induced optic neuritis indicate that patients may experience the condition unilaterally or bilaterally, with some presenting optic disk swelling. MRI findings suggest abnormalities in the optic nerve and/or the myelin sheath. The recommended treatment involves antiviral therapy combined with corticosteroid treatment, and the prognosis is uncertain, possibly associated with timely administration of medication. The pathological mechanism may be attributed to direct damage by VZV or optic nerve demyelination caused by an immune response (18). Infectious optic neuropathy induced by viruses has been extensively reported, with documented cases linked to the Chikungunya virus (CHIKV) infection during a short epidemic in 2006–2007 in Southern India. Patients may experience the condition unilaterally or bilaterally, both accompanied by optic disk swelling. Corticosteroids have been found to expedite recovery when initiated early in the disease. The possible causes may include direct viral involvement and a delayed immune response after a viral infection. A good response to corticosteroid therapy indicates the possibility of an autoimmune mechanism in the pathogenesis of the disease (18, 19).

Among ON cases caused by coronaviruses, COVID-19 ON has been most frequently documented, predominantly in the form of individual cases or case series, lacking systematic clinical presentations and treatment outcomes (5–11). In our study, all patients presented with bilateral involvement, differing from optic neuropathy induced by HIV, VZV, and CHIKV, which can affect either one or both eyes. Patients exhibited optic disk swelling similar to that observed in optic neuritis caused by CHIKV. The unique characteristics of MRI examinations in this context have not been systematically summarized in previous research. A 14-day follow-up of patients with COVID-19 ON revealed no significant difference in BCVA compared to that in the MOG-ON group; however, there was a notable improvement in the COVID-19 ON group compared to the NMO-ON group and antibody-negative ON group. At the last follow-up, BCVA in the COVID-19 ON group significantly differed from the NMO-ON group. This study suggests that patients with COVID-19 ON respond well to glucocorticoid therapy, akin to the effects observed in previous studies on corticosteroid treatment for optic neuropathy induced by CHIKV. The treatment of optic neuritis caused by HIV primarily focuses on addressing the underlying primary disease. For optic neuritis caused by VZV, the recommended approach involves a combination of antiviral therapy and corticosteroid treatment, and the prognosis remains uncertain.

Efforts to delve deeper into understanding the pathogenesis of COVID-19 ON demand heightened attention. The coronavirus, an enveloped virus with a diameter of ~120 nanometers, exhibits distinctive crown-shaped projections on its surface (20). Its genome comprises an exceptionally lengthy positive-stranded RNA. The infection process relies on the angiotensin-converting enzyme 2 (ACE2) receptor, which is present in severe acute respiratory syndrome coronavirus 1 (SARS-CoV-1) and SARS-CoV-2 and is mediated through the ACE2 receptor (21, 22). The neuropathological characteristics of COVID-19 arise from SARS-CoV-2′s direct invasion into the brain via the nasopharyngeal region. ACE2 receptors are identified in the nervous system, including glial cells and the basal ganglia (23). Inflammatory mediators disrupt the blood–brain barrier, activate glial cells, and induce demyelination of nerve fibers (24). In a previous study, it was revealed that molecular mimicry and the production of autoantibodies may contribute to immune-mediated damage to nervous tissues (23, 24). In our study, patients exhibited a favorable response to steroid treatment, suggesting that the pathological mechanism may be primarily attributed to molecular mimicry and the production of autoantibodies, contributing to immune-mediated damage to nervous tissues. This mechanism is similar to the pathogenesis of optic neuropathy induced by CHIKV. However, optic neuropathy caused by HIV and VZV may involve direct damage to the optic nerve, with or without demyelination, triggered by an immune response.

Among NMO-ON patients, 47% had a history of viral infection (25), while among MOG-ON patients, 37.5–60% had a history of previous infections (26). However, these patients were diagnosed with NMO-ON or MOG-ON rather than infectious optic neuropathy. Extensive research has been conducted on NMO-ON and MOG-ON, with standardized diagnostic and treatment guidelines and systematic summaries of clinical manifestations and MRI examinations. In alignment with this finding, we have summarized the clinical characteristics of COVID-19 ON. In comparison to NMO-ON and MOG-ON, COVID-19 ON mainly presents with bilateral onset and is more prone to optic disc edema. Previous studies have indicated that NMO-ON often involves the posterior and frequently longitudinally extensive optic nerve, with a tendency to affect the chiasmal region (14); MOG-ON is frequently longitudinally extensive and involves the optic nerve sheath (15). Our study corroborates these findings but uniquely identifies COVID-19 ON as presenting with long-segment enhancement without involvement of the intracranial segment of the optic nerve in T1-enhanced images, which is a novel observation (Figure 1).

Unfortunately, due to the retrospective nature of this relatively small sample size and short-term follow-up, our statistical conclusions lack the strongest evidence support.

## Conclusion

Our investigation highlights the favorable impact of glucocorticoid therapy on COVID-19 ON. Notably, our orbital MRI observations unveiled a unique long-segment enhancement in T1-enhanced images, excluding the involvement of the intracranial optic nerve segment. These distinctive features identified in COVID-19 ON contribute significantly to advancing our comprehension of infectious optic neuropathies, offering valuable insights for future research and clinical considerations.

## Data availability statement

The original contributions presented in the study are included in the article/supplementary material, further inquiries can be directed to the corresponding author.

## Ethics statement

Ethical review and approval was not required for the study on human participants in accordance with the local legislation and institutional requirements. Written informed consent from the patients/participants or patients/participants' legal guardian/next of kin was not required to participate in this study in accordance with the national legislation and the institutional requirements.

## Author contributions

F-FZ: Writing – original draft, Writing – review & editing. YW: Writing – review & editing. T-PL: Writing – review & editing. SH: Writing – review & editing. X-SY: Writing – review & editing. XL: Writing – review & editing. JC: Writing – review & editing. KH: Writing – review & editing. HL: Writing – review & editing. J-FY: Writing – review & editing. LC: Writing – review & editing. L-PC: Writing – review & editing.
